# Cimicifugoside H-2 as an Inhibitor of IKK1/Alpha: A Molecular Docking and Dynamic Simulation Study

**DOI:** 10.3390/biom14070860

**Published:** 2024-07-17

**Authors:** Shahd Aboul Hosn, Christina El Ahmadieh, Sergio Thoumi, Aia Sinno, Charbel Al Khoury

**Affiliations:** 1 Department of Natural Sciences, School of Arts and Sciences, Lebanese American University, Beirut Campus, P.O. Box 13-5053, Chouran, Beirut 1102 2801, Lebanonchristina.alahmadieh@lau.edu (C.E.A.);; 2 Department of Computer Science and Mathematics, Lebanese American University, Beirut Campus, P.O. Box 13-5053, Chouran, Beirut 1102 2801, Lebanon

**Keywords:** IKK1/alpha or IKK1/α, NF-κB pathway, Cimicifugoside H-2, molecular docking, dynamic simulation

## Abstract

One of the most challenging issues scientists face is finding a suitable non-invasive treatment for cancer, as it is widespread around the world. The efficacy of phytochemicals that target oncogenic pathways appears to be quite promising and has gained attention over the past few years. We investigated the effect of docking phytochemicals isolated from the rhizomes of the Cimicifuga foetida plant on different domains of the IκB kinase alpha (IKK1/alpha) protein. The Cimicifugoside H-2 phytochemical registered a high docking score on the activation loop of IKK1/alpha amongst the other phytochemicals compared to the positive control. The interaction of the protein with Cimicifugoside H-2 was mostly stabilized by hydrogen bonds and hydrophobic interactions. A dynamic simulation was then performed with the Cimicifugoside H-2 phytochemical on the activation loop of IKK1/alpha, revealing that Cimicifugoside H-2 is a possible inhibitor of this protein. The pharmacokinetic properties of the drug were also examined to assess the safety of administering the drug. Therefore, in this in silico study, we discovered that the Cimicifugoside H-2 phytochemical inhibits the actively mutated conformation of IKK1/alpha, potentially suppressing the nuclear factor kappa light chain enhancer of activated B cells (NF-κB) pathway.

## 1. Introduction

Cancer stands as a preeminent cause of mortality across more than a hundred nations [1]. The annual toll of cancer-related deaths escalated to an alarming ten million in the year 2020 [2]. In response, a multitude of targeted cancer therapeutics have emerged, meticulously engineered to modulate distinct tumorigenic pathways involved in cell cycle progression, metastasis, angiogenesis, and apoptosis. Precise drug–compound interactions orchestrate the downregulation of the aberrant signaling cascades that lead to oncogenesis [3]. While conventional therapies like immunotherapy, radiotherapy, and chemotherapy are used to treat cancer, they often result in undesirable consequences. These may encompass the development of acquired resistance, severe side effects, and suboptimal therapeutic responses [4]. Conversely, targeted therapeutics target malignant cells with pinpoint precision, sparing normal counterparts the perils of collateral damage. This finely tuned selectivity holds the promise of attenuating toxicity and curbing off-target effects, proclaiming an era of more refined cancer management [5].

The NF-κB family of transcription factors plays a pivotal role in various cellular processes, including cell growth, differentiation, and survival. However, when these factors become persistently active, they can lead to various health issues, including cancer [6,7]. This family includes five major transcription factors: p65 (Rel A), Rel B, c-Rel, p50, and p52, each contributing to the regulation of the NF-κB pathway [8,9]. These factors share a common Rel homology domain (RHD) at their N-terminus, which contains a nuclear localization signal. This domain facilitates interaction with inhibitory proteins like IκBα, IκBβ, and IκBε, keeping the transcription factors in the cytoplasm [9]. The NF-κB pathway operates through two main routes: the canonical pathway and the non-canonical pathway. In contrast to the non-canonical pathway, the canonical pathway relies on the NF-κB essential modulator (NEMO) for its activation. NEMO, acting as a regulatory subunit, forms a complex with catalytic subunits IKK1/α and IκB kinase beta (IKK2/β) to create the inhibitor of nuclear factor-κB kinase (IKK) complex. This complex phosphorylates and activates the IκB substrate, leading to the ubiquitination and subsequent degradation of IκB. Consequently, NF-κB, often in the form of a p50:Rel A heterodimer, translocates into the nucleus, affecting the transcription of different genes [8,10]. Various receptors control the activation of the canonical pathway, including pattern recognition receptors (PRRs), T cell receptors (TCR), and proinflammatory cytokine receptors [7]. On the other hand, the non-canonical pathway is triggered by receptors like the lymphotoxin beta receptor (LTβR) and B-cell activating factor receptor (BAFF) [11]. The recruitment of NF-κB inducing kinase (NIK) is a crucial step in initiating the non-canonical pathway [12]. Following this, NIK initiates the phosphorylation and activation of IKK1/α, leading to the processing of the precursor protein p100. Phosphorylated p100 undergoes partial proteasomal degradation and ubiquitination, transforming into p52 [13,14]. As a result, a p52:Rel B heterodimer complex forms and translocates into the nucleus, where it functions as a transcription factor [15]. This heterodimer complex triggers the expression of genes within the NF-κB pathway that are crucial for lymphoid organogenesis and B-cell proliferation [13]. In certain scenarios, the non-canonical pathway can stimulate tumor progression and metastasis. Consequently, targeting this pathway presents a valuable therapeutic approach for inhibiting the growth and metastasis of specific cancer subtypes, such as lymphomas and multiple myelomas [7].

It is worth highlighting the presence of crosstalk between these two pathways, signifying an interplay between inflammatory and developmental stimuli [13]. This intricate interaction underlines the significance of targeting specific hallmark proteins within oncogenic pathways to mitigate tumorigenesis while minimizing off-target effects. IKK1/α operates as a serine/threonine kinase, typically forming dimers. Occasionally, it assembles into a trimeric complex of dimers, which can then arrange into a hexamer—though the latter configuration is uncommon due to its limited stability within cells [16]. This protein encompasses three primary domains: the kinase domain (KD), the ubiquitin-like domain (ULD), and the scaffold dimerization domain (SDD). The KD regulates downstream IκB protein phosphorylation and degradation, thereby activating the NF-κB pathway. Within it resides an activation loop composed of three residues—S176, T179, and S180—where activation occurs, primarily by upstream proteins like NIK in the non-canonical pathway. One notable mutant form of IKK1/α involves a mutation of serine residues 176 and 180 to glutamate, resulting in the protein’s constitutive activation [16,17,18]. Additionally, the ATP-binding domain modulates the activation of the kinase itself [18,19]. Meanwhile, the SDD coordinates IKK1/α dimerization, forming either homodimers or heterodimers [16]. Concurrently, the ULD regulates kinase activity and guides subcellular localization by facilitating the release of NF-κB upon IκB protein phosphorylation [20]. This SDD encompasses two pivotal motifs: the leucine zipper (LZ) and helix-loop-helix (HLH). The former prominently facilitates dimerization, particularly with IKK2/β in the canonical pathway, and helps regulate gene expression by enhancing the binding of homodimers or heterodimers to specific DNA sequences. Conversely, the latter drives protein–protein interactions, including the interaction of IKK1/α with regulatory proteins [9,21,22,23]. In the non-canonical pathway, IKK1/α forms independent homodimers that are separate from the broader IKK complex seen in the canonical pathway [9]. Consequently, inhibiting IKK1/α presents a potential avenue to suppress the non-canonical NF-κB pathway, thereby reducing tumor growth and cancer cell survival.

The constitutive activation of either the canonical or non-canonical pathways could result in oncogenesis, autoimmune disease, or even chronic inflammation [24,25]. Therefore, suppressing the NF-κB pathway by inhibiting IKK1/α could lead to inhibition of autoimmune disease, inflammation, and progression of cancer [26]. Previous research has set the ground for targeting the NF-κB pathway, specifically IKK1/α. IKK1/α contains various targetable domains, including the kinase domain, NEMO-binding domain, scaffold-dimerization domain, and ubiquitin-like domain. Chrysin, a polyphenolic flavonoid compound, exhibited a remarkable affinity for the ATP-binding pocket of IKK1/α, resulting in the inhibition of the NF-κB pathway [27]. In a parallel study, the allosteric inhibition of IKK1/α outside the ATP-binding pocket was achieved through a newly synthesized glucosamine derivative drug known as NAPA [28]. Furthermore, NDSM253 emerged as a potential inhibitor of IKK1/α, demonstrating the ability to mitigate downstream signaling and foster bone healing by reducing inflammatory responses [29].

*Cimicifuga foetida*, an indigenous medicinal plant from China, boasts an array of phytochemicals found within its rhizomes [30]. This botanical repository includes notable compounds like Actein, 26-Deoxyactein, Soulieoside A, 3’-O-acetylactein, Daucosterol, Cimigenoside 25-acetate, Cimigenoside, Cimiside A, Cimicifugoside H-2, KHF16, Cimigenol-3-one, β-sitosterol, Isoferulic acid, Norkhellol, 25-O-Acetylcimigenol, Noreugenin, Cimigenol, Bergenin, and Cimifugin [30,31,32]. These compounds have garnered attention for their diverse attributes, spanning anti-tumorigenic, anti-inflammatory, and anti-viral effects [33]. Extensive exploration has focused on the anti-cancer properties of the phytochemical constituents of *C. foetida*. These agents, through various mechanisms, exert a tumoricidal influence by either obstructing DNA damage or impeding the replication of premalignant cells characterized by compromised DNA integrity [34].

A captivating dimension of this botanical resource lies within its ethyl acetate fraction—a reservoir housing 21 triterpene glycosides, among which are phenolic acids such as ferulic acid and isoferulic acid. This distinctive fraction has been implicated in initiating caspase-dependent apoptosis—a cellular process renowned for its fundamental role in programmed cell death regulation [35]. In the context of cancer therapeutics, KHF16 has emerged as a noteworthy candidate by exhibiting inhibitory effects on Triple-Negative Breast Cancer (TNBC) through the downregulation of the NF-κB pathway [32]. Furthermore, both actein and 26-deoxyactein have demonstrated anti-tumor efficacy in vitro and in vivo across a spectrum of 12 distinct cancer cell lines [36]. Additionally, noteworthy findings have emerged regarding the potential of the medicinal extract of *C. foetida* to mitigate menopause-related symptoms, offering a promising avenue for addressing this health concern [37].

Hence, our study embarked on an exploration of the potential inhibitory impact of phytochemicals extracted from *C. foetida* on the tumorigenic NF-κB pathway. To achieve this, we employed molecular docking and dynamic simulation methodologies to foresee binding free energy estimations and unveil interactions between the phytochemicals and IKK1/α.

## 2. Materials and Methods

### 2.1. Preparation of the Ligand

The 3D structure of the ligands was retrieved from the PubChem database [38] in .sdf format and subsequently converted to .pdbqt format using Open Babel [39]. For some ligands, the structure was drawn using ICM Pro (https://www.molsoft.com/) accessed on 4 September 2023.

### 2.2. Preparation of the Protein

Utilizing the RCSB PDB [40], the 3D structure of the mutated IKK1/α (PDB ID: 5EBZ) hexamer was retrieved in .pdb format. Next, the protonation states of the residues were determined from the results obtained with the PDB2PQR server [41] . Subsequently, the structure was then converted to .pdbqt format using Open Babel. This protein model was introduced into the Autodock 1.5.7 software [42] and refined by the exclusion of water molecules. Docking procedures were executed on an IKK1/α monomer.

The crystal structure (.pdb format) of the protein was also imported into Molsoft ICM-Pro v3.9-1b [43]. Water molecules present in the X-ray structures were removed. Hydrogens were optimized to find the best hydrogen bonding network, and critical amino acids such as histidine, proline, asparagine, glycine, and cysteine were optimized for the best orientation and protonation state. Missing side chains were treated before setting the protein for docking.

### 2.3. Molecular Docking

#### 2.3.1. Docking with AutoDock

First, we performed molecular docking using the Autodock software; the protein and ligands were imported into AutoDock, whereby polar hydrogen atoms and Kollman charges were included in the protein configuration. Correspondingly, the ligands underwent a process involving the introduction of Gestgeiger charges and the establishment of torsions. Subsequently, the Lamarckian Genetic Algorithm was applied to explore the interaction between the ligand and the binding site of the protein. The simulation was conducted over a span of 10 runs. The results were then subjected to ranking based on their binding affinity. Notably, the docking methodology utilized in this study aims to predict the change in free energy upon ligand binding. Therefore, a higher binding affinity between the protein and the ligand translates to a lower binding energy. Consequently, more negative values in the binding energy indicate a stronger binding affinity.

#### 2.3.2. Docking with ICM-Pro

To validate our results, we also performed docking using Molsoft ICM-Pro v3.9-1b. After the protein preparation steps mentioned above, the binding site residues of the viral proteins were docked with the ligands. At the target site, hydrogen bonding potential, van der Waals potential (with carbon-, sulfur-, and hydrogen-like probes), hydrophobic potential, and electrostatic potential were considered. The conformational search in ICM-Pro utilized the Biased Probability Monte Carlo (BPMC) method, which randomly selects poses in the internal coordinate space and makes steps to new random positions independent of the previous ones, according to a predefined constant probability distribution. For this study, the thoroughness, representing the length of the simulation, was set to 10. The ligand conformations were ranked using the ICM score, with more negative scores indicating stronger binding of the protein–ligand complex.

### 2.4. Molecular Dynamic Simulation

Molecular dynamics (MD) offers the capability to predict crucial structural and thermodynamic alterations within the IKK1/α-Cimicifugoside H-2 complex. The docking poses of Cimicifugoside H-2 in relation to IKK1/α were generated through AutoDock. Subsequently, an active conformation of IKK1/α was established, characterized by two mutations, S176E and S180E, and employed for MD simulations using the GROningen Machine for Chemical Simulations with the GPU-enabled package (GROMACS 2020.4). The simulations were conducted at a neutral pH. The CHARMM36 force field in GROMACS format (.gro) was adopted, obtained from the MacKerell lab website (http://mackerell.umaryland.edu/charmm_ff.shtml; accessed on 6 September 2023). The protein–ligand complex was enclosed within a dodecahedral cell-shaped unit and solvated using a single point charge (SPC) water model. To maintain a neutral system, the addition of four sodium ions was carried out using the “gmx genion” script. For energy minimization of the complex, the steepest descent algorithm was implemented, ensuring energy reaching below 10 kJ/mol [44]. This process resulted in the reduction of pressure and temperature. The V-rescale thermostat was employed to maintain a constant temperature of 300 K, while the Parrinello–Rahman barostat was used to maintain a constant pressure of 1 bar during the NPT equilibration and production MD phases. The particle mesh Ewald (PME) method was employed to account for long-range electrostatic interactions, with Fourier spacing and cut-off distances set at 12 Å. Electrostatic and van der Waals interactions were taken into consideration within a 1 nm cut-off range. The bonds of all heavy atoms were constrained through the LINCS algorithm. The equilibration phase consisted of two steps: 100 ps under NVT conditions (constant number of particles, volume, and temperature) followed by 100 ps under NPT conditions (constant number of particles, pressure, and temperature). A dynamic simulation was then conducted on the equilibrated system over a span of 100 ns, utilizing the leapfrog method [45] under NPT ensemble conditions. Trajectories from the MD simulation were analyzed through distinct GROMACS scripts, enabling the computation of parameters such as Root Mean Square Deviation (RMSD), Root Mean Square Fluctuation (RMSF), radius of gyration (Rg), and peptide–peptide hydrogen bonding [46].

### 2.5. Structural Alignment and RMSD Calculation

The best docking pose of the ligand and the last frames (96, 97, 98, 99, and 100 ns) of the MD simulation were loaded into ICM-Pro. The structures were aligned using the superimpose function in ICM-Pro, focusing on the protein backbone atoms (Cα atoms). RMSD values were calculated to quantify the structural differences between the docking pose and the MD simulation poses.

### 2.6. Pharmacokinetic Properties

We investigated the pharmacokinetic attributes of Cimicifugoside H-2 through the utilization of SwissADME [47] and pkCSM [48]. These readily accessible resources furnished comprehensive insights into the characteristics of the drug pertaining to absorption, distribution, metabolism, excretion, and toxicity.

### 2.7. Network Pharmacology

#### 2.7.1. Pathway Enrichments Analysis

We focused our analysis on significantly enriched pathways. The pathway enrichment analysis was performed using Enrichr, specifically selecting the MSigDB Hallmark 2020 pathway [13]. Enrichr integrates various gene set libraries and allows for the identification of enriched pathways based on the input gene we used, which was IKK1/α, also known as CHUK.

#### 2.7.2. Protein–Protein Interactions

The protein–protein network interactions were carried out by referencing the STITCH database [2]. The interactions were computed based on the known profiles and interaction tendencies of the IKK1/α protein.

## 3. Results

### 3.1. Molecular Docking on IKK1/α

We conducted molecular docking assessments involving 19 diverse phytochemicals extracted from *C. foetida* against four discrete domains of IKK1/α. For benchmarking, a small molecular weight inhibitor termed 2-azanyl-5-phenyl-3-(4-sulfamoylphenyl)benzamide was employed as a positive control [40]. The binding affinities of the phytochemicals were evaluated and compared with those of the positive control (Table 1).

#### 3.1.1. Activation Loop

The activation loop comprises three key residues, namely S176, T179, and S180 [15,18]. When the phytochemicals were subjected to docking within this domain of IKK1/α, consisting of a double mutant S176E and S180E, robust binding affinities were observed. Particularly noteworthy was the interaction of Cimicifugoside H-2, which exhibited a notable binding energy of −10.22 and −10.17 kcal/mol compared to the positive control, −7.81 and −7.86 kcal/mol, using AutoDock and ICM-pro, respectively.

#### 3.1.2. ATP-Binding Domain

The ATP-binding domain extends from residues 21–29 [24]. The same phytochemical, which is Cimicifugoside H-2, registered the highest binding affinity to the ATP-binding domain with similar binding energies of −10.22 kcal/mol using AutoDock and −10.21 using ICM-pro.

#### 3.1.3. Helix-Loop-Helix Motif

The HLH motif extends from residues 599–638 [9]. Actein, with a binding energy of −10.95 and −10.84 kcal/mol, using AutoDock and ICM-pro, respectively, was the phytochemical with the highest binding affinity to this domain.

#### 3.1.4. Leucine Zipper Motif

The LZ motif consists of residues 455–476 [24]. The phytochemical with the greatest binding affinity was 25-O-Acetylcimigenol, at −9.31 (AutoDock) and −9.31 (ICM-pro) kcal/mol.

### 3.2. Molecular Docking on IKK2/β

IKK1/α and IKK2/β exhibit a significant 65% identity in their catalytic domains. Moreover, their homology remains notably high, reaching approximately 52% [16,44]. Given this substantial similarity, our investigation led us to undertake docking studies of the phytochemical Cimicifugoside H-2, which displayed the highest binding energy compared to the positive control, onto the activation loop of mutated IKK2/β. This endeavor aimed to reveal the specificity of this phytochemical. The activation loop of the mutated IKK2/β encompasses two key residues, S177E and S181E [22]. The binding energy computed for the activation loop of IKK2/β was −5.1 kcal/mol.

**Figure 1:**
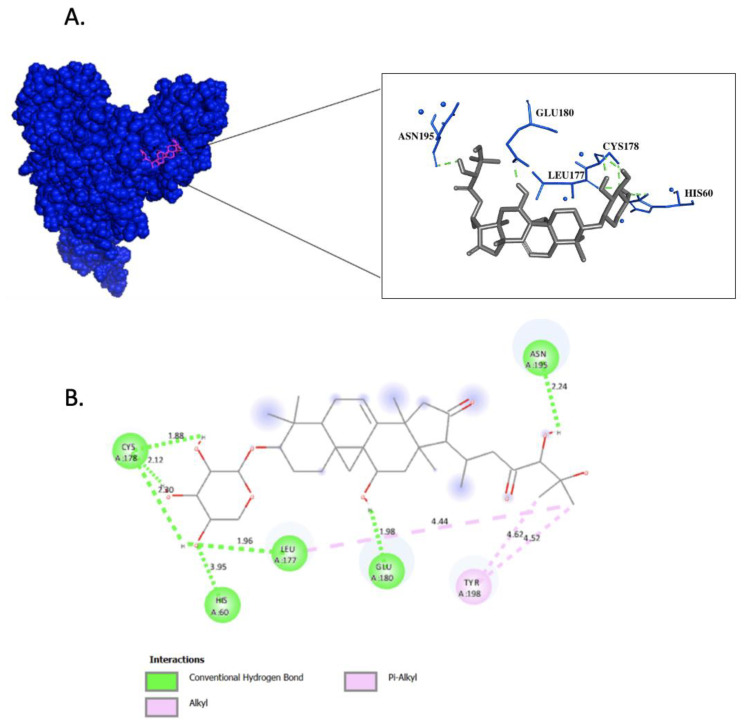
Molecular Docking of Cimicifugoside H-2 on IKK1/α. (**A**) Surface representation of IKK1/α protein in blue and Cimicifugoside H-2 in magenta showing the interacting residues. (**B**) 2D diagram of protein–ligand interaction; hydrogen bonding is represented in green, and hydrophobic interactions are represented in pink.

We conducted a thorough analysis of the hydrogen bonding and hydrophobic interactions that underlie the binding of Cimicifugoside H-2 to IKK1/α, as depicted in Figure 1. The presence of seven hydrogen bonds, of which six exhibited robust stability, characterized by distances <3.5 Å, was revealed [49]. Specifically, H60 participated in hydrogen bond formation at a distance of 3.95 Å (Hbond 1); L177 engaged in hydrogen bond at a proximity of 1.96 Å (Hbond 2); C178 formed three distinct hydrogen bonds at distances of 1.88 (Hbond 3), 2.12 (Hbond 4), and 2.30 Å (Hbond 5); E180 contributed to hydrogen bond formation at a distance of 1.98 Å (Hbond 6); and N195 established a hydrogen bond at a distance of 2.24 Å (Hbond 7). Furthermore, noteworthy hydrophobic interactions between the ligand and two residues, L177 and Y198, bear significance due to their occurrence at distances surpassing 3.8 Å, as elucidated by previous studies [50].

### 3.3. Dynamic Simulation

**Figure 2:**
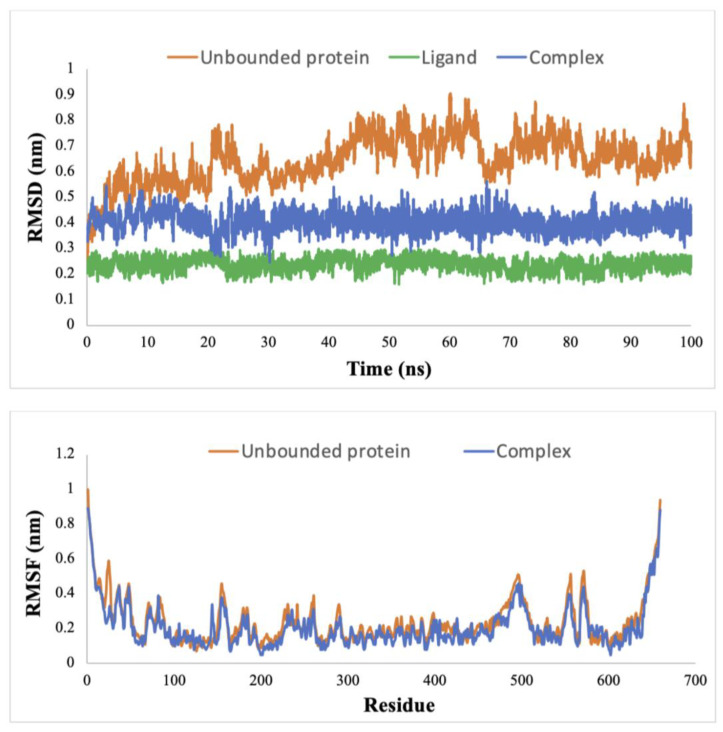
Root Mean Square Deviation (RMSD) of IKK1/α protein in complex with Cimicifugoside H-2 during 100 ns of the molecular dynamic simulation period and Root Mean Squared of Fluctuation (RMSF) of IKK1/α protein both as unbound and in a complex with Cimicifugoside H-2.

We performed an MD simulation to explore the effects of docking Cimicifugoside H-2 onto the activation loop of IKK1/α, aiming to uncover the structural alterations [51]. The dynamic simulation showcased the enduring stability of Cimicifugoside H-2 throughout its complex formation with IKK1/α. The RMSD value of the protein–ligand complex was 0.38 nm, as shown in Figure 2. We also analyzed the ligand positional RMSD to predict its binding stability to the protein. Intriguing insights emerged as we plotted the RMSF value against the 660 residues of the mutated, constitutively active IKK1/α. Notably, we observed diverse fluctuations among residues, specifically within its kinase domain (Figure 2).

**Figure 3:**
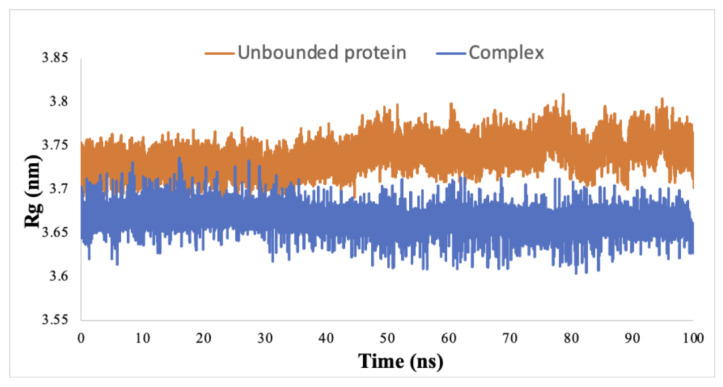
Radius of Gyration (Rg) of IKK1/α protein both as unbound and in a complex with Cimicifugoside H-2.

In fact, residues 21–29 and 144 displayed marked variations in the RMSF value, distinguishing the complex-bound state from the unbound protein and pointing towards the structural flexibility of these amino acids. Other amino acids also exhibited more modest flexibility, such as the residues 176E, 179T, and 180E, which constitute the activation loop. Moreover, the Rg analysis provided insights into the compactness of the protein upon ligand binding. The Rg value for the unbound protein remained constant at 3.72 nm. In contrast, when the ligand interacted with the protein, the Rg value decreased to 3.66 nm and maintained this compact state throughout the simulation (Figure 3).

Interestingly, the hydrogen bonding pattern of the complex emerged during the 100 ns simulation, revealing a consistent average of five to seven hydrogen bonds formed between the protein and the ligand, further emphasizing the stability of the complex over the duration of the simulation (Table 2).

Utilizing MM/PBSA (Molecular Mechanics/Poisson–Boltzmann surface area)-based calculations, we quantified the enthalpy within the IKK1/α-Cimicifugoside H-2 complex. The resultant binding free energy was calculated to be −187.17 kJ/mol. This substantial binding affinity was primarily attributed to the van der Waals interactions (Table 3).

### 3.4. Conformational Consistency in Molecular Docking and Dynamic Simulation

The structural alignment and RMSD analysis revealed highly similar structures with minimal deviations between the binding pose predicted by docking and the poses observed in the last frames of the MD simulation. Low RMSD values for the alignment of the docking pose with different frames of the last 5 ns of the MD simulation were detected (Table 4). These results are in line with the RMSD of the ligand recorded along the course of the trajectory (Figure 2), further supporting the idea that the ligand experienced minimal fluctuations during the MD simulation.

To evaluate the consistency of hydrogen bond interactions between docking and MD simulations, we analyzed the hydrogen bonds formed between the protein and ligand across the five MD simulation frames. Our analysis revealed that, except for HBond 1, all other hydrogen bonds identified in the docking pose were consistently observed throughout the MD simulation frames. This indicates a strong agreement between the docking predictions and the dynamic behavior of the protein–ligand complex, reinforcing the importance of these critical hydrogen bonds in maintaining the binding pose.

### 3.5. Pharmacokinetic Properties

To delve deeper into the mechanism underpinning the action of Cimicifugoside H-2, a comprehensive investigation of its molecular attributes (Table 5) and pharmacokinetic characteristics (Table 6) was undertaken. With a low molecular weight of 634.8 g/mol and a precisely calculated logarithm of partition coefficient (logP) value of 2.0467 (Table 5), Cimicifugoside H-2 exhibits a limited gastrointestinal (GI) absorption and a notable inability to traverse the blood–brain barrier (BBB) (Table 6). Furthermore, Cimicifugoside H-2 appears to be a substrate of P-glycoproteins and CYP3A4 (cytochrome P3A4). Its total clearance is somewhat low; however, the drug exhibits no toxicity in appropriate doses in the body and is non-carcinogenic (Table 6).

### 3.6. Network Pharmacology

**Figure 4:**
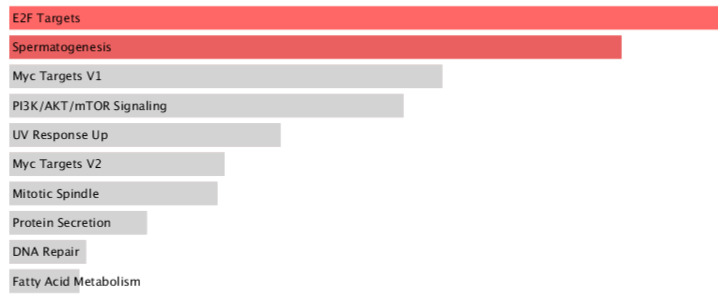
Top 10 significant Enrichr MSigDB Hallmark 2020 proteins. The significantly expressed proteins have *p* ≤ 0.05. The brightness and the length of the bar represent the significance of the category/term.

The pathway enrichment analysis revealed several key pathways that could possibly be affected by the knockdown of IKK1/α. The analysis was based on the *p*-value of each pathway. The smaller the *p*-value, the greater the significance that a specific pathway would be affected by the inhibition of IKK1/α by our drug. The two pathways that showed significance were the E2F and spermatogenesis pathways (Figure 4).

**Figure 5:**
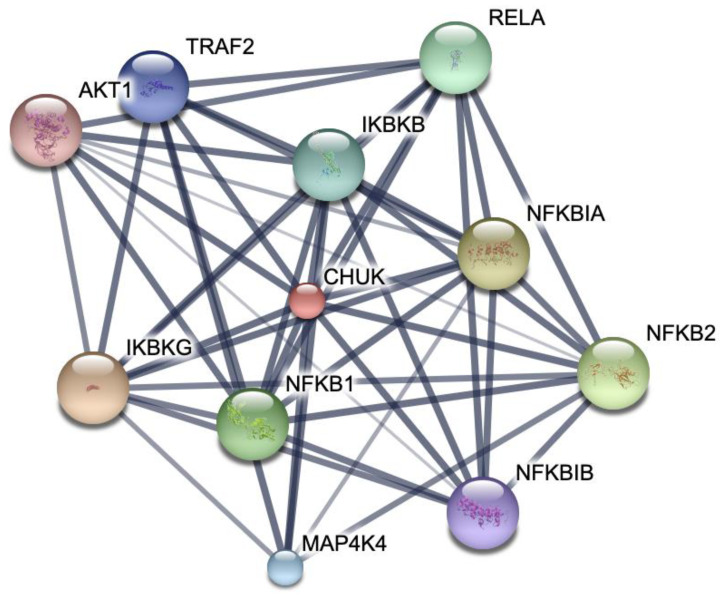
Protein–protein interaction network of IKK1/α.

We further elucidated the possible protein–protein interactions that may arise with IKK1/α being our protein of interest. Several interactions with a high confidence score were noted using the STRING database, revealing the presence of multiple clusters (Figure 5).

## 4. Discussion

**Figure 6:**
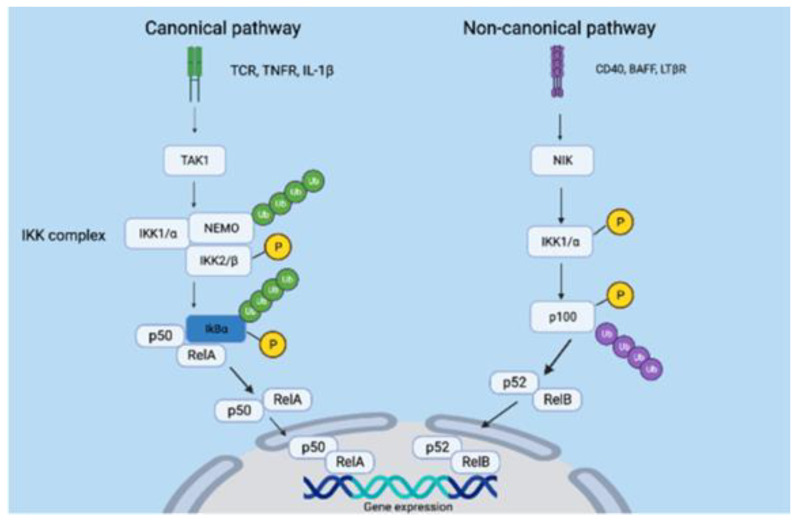
The canonical and non-canonical NF-κB pathways. The canonical pathway is activated by a wide range of receptors, such as the T-cell receptor (TCR), tumor necrosis factor receptor (TNFR), and interleukin-1 beta (IL-1β). The receptors recruit multiple proteins to activate the transforming growth factor β−activatedkinase1 (TAK1). TAK1 then mediates the phosphorylation and activation of IKK2/β, making it available to associate with IKK1/α and NEMO. This IKK complex then targets the substrate IkBα for polyubiquitination phosphorylation, which results in its degradation. The p50:Rel A heterodimers are then formed, and they translocate to the nucleus to affect gene expression. However, in the non-canonical pathway, receptors such as cluster of differentiation 40 (CD40), B-cell activating factor receptor (BAFF), and lymphotoxin beta receptor (LTβR), activate the nuclear factor kB-inducing kinase (NIK). NIK then phosphorylates and activates IKK1/α, which in turn process p100, leading to its polyubiquitination and degradation. This results in the formation of p52:Rel B, which promotes gene transcription in the nucleus.

Our research attempted to investigate the effect of docking various phytochemicals, extracted from the rhizomes of *C. foetida*, upon an actively mutated form of the IKK1/α protein. This mutation pertains to the substitution of serine residues within the activation loop with negatively charged glutamic acid, specifically S176E and S180E. This substitution ingeniously mimics the negative charge of a phosphate moiety, thereby leading to the constitutive activation of the IKK1/α protein, effectively bypassing the need for NIK-mediated activation [18]. The significance of targeting IKK1/α is its integral role in the NF-κB pathway, which is comprised of two pathways—the canonical and non-canonical pathways [52]. Figure 6 shows the two pathways. IKK1/α stands as the essential representative of the non-canonical pathway, facilitating the processing of p100 and triggering the formation of p52:Rel B heterodimers [13].

While various drugs have been developed to target IKK1/α, their selectivity and specificity remain challenging due to the significant homology shared between IKK1/α and IKK2/β [53]. Additionally, the conserved nature of the ATP-binding domain in certain kinases raises concerns about potential off-target effects when targeting this domain [54]. Thus, an alternative strategy could involve targeting the activation loop of IKK1/α, as this region exhibits lower conservation across kinases [55]. These investigations were carried out using a mutated form of IKK1/α composed of 660 residues [40]. Upon performing molecular docking on IKK1/α, our findings suggest that phytochemicals derived from the rhizomes of *C. foetida* hold promise for inhibiting the activation and function of IKK1/α. Notably, among these phytochemicals, Cimicifugoside H-2 displayed the highest affinity for the activation loop of IKK1/α compared to its positive control. The specific residues engaging with Cimicifugoside H-2 encompass H60, C178, L177, E180, N195, and Y198 (Figure 1). Establishing a stable interaction between the ligand and the mutated protein opens avenues for modulating a constitutively active protein like IKK1/α. To enhance specificity, we attempted to dock Cimicifugoside H-2 onto the activation loop of mutated IKK2/β, reaffirming its preference for IKK1/α. Notably, the binding affinity was characterized by a binding energy of −5.1 kcal/mol, half the energy calculated for IKK1/α. In this research paper, we have laid the foundation for targeting the activation loop of mutated IKK1/α, striving to counteract its activation mechanism driven by negative charge. Of note, the Actein phytochemical registered a higher affinity to the leucine zipper motif and helix-loop-helix motif. The mechanism of action and specificity of Cimicifugoside H-2 remains more important. Cimicifugoside H-2 inhibits the constitutively active and mutated form of IKK1/α with a high affinity to its activation loop. This allows the drug to target the intrinsic activity of the kinase, highlighting the drug’s specificity and direct inhibition of the mutated protein. Meanwhile, actein is capable of inhibiting the other motif domains of IKK1/α that are mainly responsible for initiating interactions with other proteins. IKK1/α alpha would need to dimerize to become active; yet, this inhibition might be indirect since it does not accurately target the mutation found in the activation loop of IKK1/α. Over and above, while our current study focused on the predominant forms of the ligands, we recognize the potential impact that different stereoisomeric and tautomeric forms could have on binding interactions and affinities. Future work will aim to include a comprehensive analysis of these variations to provide a more exhaustive understanding of ligand–protein interactions and to further validate the robustness of our results.

Furthermore, we conducted MD simulations to assess the stability of interactions between the protein and the drug over time and to anticipate potential conformational changes in residues beyond the activation loop. The RMSD value serves as a valuable tool for predicting structural deviations among different protein–ligand conformations [56]. The complex demonstrated a decreased RMSD value compared to the unbound protein, signifying that the protein achieved stabilization upon ligand binding. Remarkably, the RMSD value for the complex reached a state of equilibrium within the initial 20 ns of the simulation and remained consistently stable throughout the 100 ns run. Furthermore, we relied on the RMSF value to elucidate the behavior of individual residues in the protein. This parameter highlights alterations in atomic movements by contrasting the fluctuation pattern of RMSF between the protein in its bound and unbound states [57]. Residues 22-27 exhibited the most pronounced variation, notably within the ATP-binding domain of the IKK1/α protein. A noteworthy conformational alteration, particularly in T23, could potentially contribute to a reduction in phosphorylation and subsequent attenuation of kinase activity, resulting in the inhibition of the NF-κB pathway [58]. Furthermore, D144 also established significant variability in its RMSF value. This residue occupies the active site of the protein, which is presumably the location where p100 primarily binds for phosphorylation and activation [24]. Hence, Cimicifugoside H-2 demonstrates characteristics of an allosteric inhibitor, as its binding to the activation loop initiates conformational alterations in residues beyond this region. The calculated Rg value was reduced upon binding of the protein to the drug, which indicates that the protein has adopted a more compact structure. This modified conformation potentially restricts the accessibility of specific residues, impeding their initial intended functionality. In addition, an average of approximately five hydrogen bonds formed between the protein and the drug, signifying a robust and stable protein–ligand interaction. Moreover, these hydrogen bonds persisted throughout the 100 ns simulation, confirming the sustained binding of the protein to the drug and proving the maintenance of its specific orientation. Lastly, the drug exhibited a considerable binding energy to the protein, confirming its strong affinity for IKK1/α.

To confirm the feasibility of administering Cimicifugoside H-2, we studied its pharmacokinetic properties. The molecular weight of Cimicifugoside H-2 is 634.8 g/mol, and its surface area is 1.74 × 10^−18^ m^2^ (173.98 Å^2^), so it can be considered a small molecular weight inhibitor [59]. The drug would be less costly and more suitable for storage and transportation [5]. A key determinant of its lipophilicity, logP value was computed at 2.0467. Cimicifugoside H-2 is in the optimal range 0 < log P < 3; therefore, this implies that it is moderately lipophilic [60]. Its lipophilic properties serve its intracellular mode of action, as it is capable of crossing the plasma membrane. In contrast, the drug may have some off-target effects if its log *p* value is >3 [61]. The drug was also shown to be moderately soluble in water, creating a stable drug concentration in the bloodstream and promoting flexibility in formulation [62]. However, the drug has low permeability to Caco-2 cells and low GI absorption, making it somehow challenging to be administered orally. For this reason, we suggest administering the drug intravenously to ensure that it is absorbed by the cells [63]. Meanwhile, different experiments must be performed to test the permeability of this drug to different types of cells. Moreover, Cimicifugoside H-2 appears to interact as a substrate with P-glycoprotein (Pgp), a circumstance that may result in its efflux from cells [64]. This might reduce the therapeutic efficacy of the drug. Meanwhile, if a drug was shown to be a Pgp substrate, it can be administered with a Pgp inhibitor to counter the effect [65]. Furthermore, administering the drug intravenously can improve the bioavailability of the drug, which was compromised by the drug being a Pgp substrate [66]. Interestingly, Cimicifugoside H-2 assumes a dual role, serving both as a substrate and an inhibitor of Pgp, a dynamic that effectively sustains its bioavailability. It is noteworthy to mention that Cimicifugoside H-2 demonstrates restrained penetration across the BBB, thereby mitigating potential central nervous system side effects [64]. The drug also has an optimal volume of distribution that creates a balance between harmful accumulation in normal cells and sufficient amounts reaching the targeted cells [67]. There is a moderate concentration of the drug found unbound in the plasma, proving that the drug can have an effective therapeutic window without being toxic. Concerning the metabolic fate of the drug, it emerges as a substrate for the liver enzyme CYP3A4, suggesting its potential transformation into a more hydrophilic metabolite for subsequent elimination from the body [68]. Conversely, it does not exhibit substrate characteristics for the CYP2D6 liver enzyme. The drug is not an inhibitor of CYP2D6, CYP3A4, CYP1A2, CYP2C19, or CYP2C9 liver enzymes; thus, Cimicifugoside H-2 would be less likely interfere with the metabolism of other drugs by these enzymes [69]. The clearance rate of the drug is low, thus extending its duration in systemic circulation. It does, however, serve as a renal organic cation transporter 2 (OCT2) substrate to a great extent, which leads to its renal secretion [70]. Overall, Cimicifugoside H-2 demonstrates a favorable profile. Its propensity to sensitize the skin remains minimal, diminishing the likelihood of inciting skin allergies. Evidently, the compound exhibits no AMES toxicity, signifying its low mutagenic potential [71]. Moreover, it does not induce adverse effects on hepatic function. Pertinently, oral acute toxicity in rats (LD50) was calculated at 3.303 mol/kg or 2097 mg/kg, indicative of a moderate acute toxicity level. This signifies that 2097 mg/kg is a lethal dose for 50% of orally administered rats. Additional in vitro and in vivo studies must be performed to experimentally test the safety of administering this drug and understand its potential side effects.

Upon investigating the involved pathways that could be affected by the knockdown of IKK1/α, the E2F pathway appears to be mostly enriched with a *p*-value of 0.018 (significance is less than 0.05). E2F plays an important role in cell cycle progression; therefore, the results suggest that our drug could potentially halt the proliferation of cancer cells at specific cell cycle checkpoints by overexpressing E2F [7]. Moreover, IKK1/α can have an impact on spermatogenesis since it requires cell differentiation and proliferation that could possibly be induced by the kinase [71]. It also might be that the IKK1/α part of NF-κB could protect sperm cells from apoptosis and promote proliferation and differentiation of spermatogonia. Therefore, downregulation of IKK1/α could have some negative aspects that may be caused by traditional treatment methods for cancer, such as chemotherapy. Other pathways shown have a *p*-value greater than 0.05, indicating that they are less likely to be affected by the inhibition of this kinase. Although it is possible that these pathways could be affected, further experimental analyses must be performed to validate the in silico results.

We used the STRING database to investigate the possible interactions of IKK1/α with other cellular pathways. This analysis revealed that there are several network interactions that exist between IKK1/α and other proteins. The confidence score was 0.999 for all the proteins shown in Figure 6, highlighting the crosstalk of different cellular mechanisms. The interaction with several other proteins provides significant implications for understanding the mechanism of action of our drug. This could suggest that suppression of IKK1/α could possibly halt other pathways due to its wide array of interaction with other proteins, such as IKK2/β, AKT1, MAP4K4, and TRAF2, that are possibly implicated with other cancer pathways.

The integration of network pharmacology with our initial docking studies on the mutated IKK1/α protein highlights the wide array of interactions and the crosstalk of different pathways critical for cancer progression and other related diseases. These insights lay the groundwork for further experimental validation and clinical exploration, contributing to the development of targeted therapies for conditions involving the NF-κB pathway and its associated proteins. Future studies should focus on validating these in silico findings through experimental and clinical research to fully harness the therapeutic potential of the drug.

## 5. Conclusions

This study demonstrates that Cimicifugoside H-2 is a potential small molecular weight inhibitor of the NF-κB pathway that targets an actively mutated conformation of IKK1/α. Employing a comprehensive approach encompassing molecular docking, dynamic simulation, and ADMET properties, we have uncovered the underlying mechanism of action of the drug while thoroughly assessing its safety profile. To our knowledge, this is the first inhibitor that targets the activation loop of IKK1/α, making this research a novel approach. Altogether, Cimicifugoside H-2 can render IKK1/α inactive; yet, further molecular experiments, such as RT-qPCR, ought to be performed to validate our findings. To comprehensively understand the target gene’s impact on cancer, future studies will incorporate expression analysis and gene network analysis. These approaches will provide more detailed insights into the gene’s involvement in cancer progression and further validate the therapeutic potential of Cimicifugoside H-2, thereby expanding on the current findings. Over and above, such experiments can help detect phosphorylation levels of certain proteins, which is reflective of their activation to help prove that they suppress the NF-κB pathway. Finally, the safety of the drug must be further examined as it moves from the drug discovery process to enter pre-clinical and clinical studies. Therefore, Cimicifugoside H-2 can serve as a lead compound to synthesize other potent IKK1/α inhibitors, paving the way for the development of new cancer therapeutics.

## Figures and Tables

**Table 1:** Binding energies of the phytochemicals isolated from rhizomes of *C. foetida* against IKK1/α protein.

Domain	Drug	AutoDock Binding Energy (kcal/mol)	ICM-Pro ICM Score (kcal/mol)
Activation loop	Positive control	−7.81	−7.86
	Actein	−5.87	−5.69
	26-Deoxyactein	−4.25	−4.18
	Soulieoside A	−4.9	−4.95
	3′-O-acetylactein	−9.59	−9.64
	Daucosterol	−7.72	−7.71
	Cimigenoside,25-acetate	−6.06	−6.09
	Cimigenoside	−6.71	−6.82
	Cimiside A	−5.38	−5.46
	Cimicifugoside H-2	−10.22	−10.17
	KHF16	−6.58	−6.44
	Cimigenol-3-one	−6.6	−6.42
	Beta-sitosterol	−6.3	−6.24
	Isoferulic acid	−6.71	−6.72
	Norkhellol	−6.55	−6.49
	25-O-Acetylcimigenol	−4.9	−4.93
	Noreugenin	−6.57	−6.75
	Cimigenol	−6.71	−6.77
	Bergenin	−8.36	−8.34
	Cimifugin	−7.36	−7.39
ATP-binding domain	Positive control	−8.34	−8.27
	Actein	−9.08	−9.15
	26-Deoxyactein	−9.82	−9.92
	Soulieoside A	−8.18	−8.22
	3′-O-acetylactein	−7.74	−7.74
	Daucosterol	−9.9	−9.88
	Cimigenoside,25-acetate	−9.32	−9.3
	Cimigenoside	−7.58	−7.61
	Cimiside A	−8.53	−8.55
	Cimicifugoside H-2	−10.22	−10.21
	KHF16	−8.67	−8.68
	Cimigenol-3-one	−7.39	−7.42
	Beta-sitosterol	−6.97	−7.02
	Isoferulic acid	−8.16	−8.11
	Norkhellol	−9.34	−9.37
	25-O-Acetylcimigenol	−9.11	−9.07
	Noreugenin	−8.85	−8.83
	Cimigenol	−7.73	−7.73
	Bergenin	−8.97	−8.95
	Cimifugin	−9.61	−9.6
Helix-loop-helix motif	Positive control	−9.34	−9.41
	Actein	−10.95	−10.84
	26-Deoxyactein	−9.48	−9.44
	Soulieoside A	−9.26	−9.18
	3′-O-acetylactein	−8.7	−8.61
	Daucosterol	−8.25	−8.29
	Cimigenoside,25-acetate	−10.05	−10.16
	Cimigenoside	−9.74	−9.73
	Cimiside A	−9.23	−9.18
	Cimicifugoside H-2	−8.54	−8.52
	KHF16	−8.55	−8.57
	Cimigenol-3-one	−7.44	−7.45
	Beta-sitosterol	−7.4	−7.48
	Isoferulic acid	−7.17	−7.12
	Norkhellol	−7.56	−7.52
	25-O-Acetylcimigenol	−9.44	−9.41
	Noreugenin	−7.94	−7.92
	Cimigenol	−7.99	−7.93
	Bergenin	−8.51	−8.54
	Cimifugin	−8.05	−8.11
Leucine zipper motif	Positive control	−7.85	−7.81
	Actein	−9.18	−9.15
	26-Deoxyactein	−8.52	−8.55
	Soulieoside A	−8.51	−8.52
	3′-O-acetylactein	−7.49	−7.38
	Daucosterol	−7.81	−7.88
	Cimigenoside,25-acetate	−8.95	−8.81
	Cimigenoside	−7.94	−7.89
	Cimiside A	−7.32	−7.25
	Cimicifugoside H-2	−7.59	−7.64
	KHF16	−7.35	−7.44
	Cimigenol-3-one	−6.72	−6.68
	Beta-sitosterol	−6.19	−6.09
	Isoferulic acid	−5.41	−5.33
	Norkhellol	−6.13	−6.11
	25-O-Acetylcimigenol	−9.31	−9.34
	Noreugenin	−5.9	−5.91
	Cimigenol	−6.36	−6.33
	Bergenin	−7.29	−7.34
	Cimifugin	−6.18	−6.25

**Table 2:** Comparison of hydrogen bonds in molecular docking and molecular dynamics simulation.

Residues Establishing Hydrogen Bonds	Molecular Docking	Molecular Dynamics Simulation	Percentage in Molecular Dynamics Simulation (%)
H60 (Hbond 1)	Yes	Yes	22.6
L177 (Hbond 2)	Yes	Yes	100
C178 (Hbond 3)	Yes	Yes	100
C178 (Hbond 4)	Yes	Yes	94.9
C178 (Hbond 5)	Yes	Yes	84.3
E180 (Hbond 6)	Yes	Yes	92.57
N195 (Hbond 7)	Yes	Yes	86.7

**Table 3:** Average enthalpy calculated from the molecular dynamics simulation in triplicate.

Protein–Ligand Complex	Van der Waals (kJ/mol)	Electrostatic (kJ/mol)	Polar Energy (kJ/mol)	Non-Polar Energy (kJ/mol)	Enthalpy (kJ/mol)
	−127.32 ± 3.3	−11.29 ± 0.4	−35.48 ± 0.6	−13.08 ± 0.3	−187.17 ± 3.7

**Table 4:** RMSD values and hydrogen bonds comparison between docking and molecular dynamics simulation poses.

Frame (ns)	RMSD (Å)	Hydrogen Bond
96	0.09	Hbond 2, Hbond 3, Hbond 4, Hbond 5, Hbond 6, and Hbond 7
97	0.1	Hbond 2, Hbond 3, Hbond 4, Hbond 5, Hbond 6, and Hbond 7
98	0.09	Hbond 2, Hbond 3, Hbond 4, Hbond 5, Hbond 6, and Hbond 7
99	0.11	Hbond 2, Hbond 3, Hbond 4, Hbond 5, Hbond 6, and Hbond 7
100	0.12	Hbond 2, Hbond 3, Hbond 4, Hbond 5, Hbond 6, and Hbond 7

**Table 5:** Molecular characteristics of Cimicifugoside H-2.

2D Representation	SMILES	Molecular Weight (g/mol)	LogP	Surface Area (m^2^)
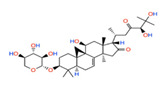	CC(CC(=O)C(C(C)(C)O)O)C1C(=O)CC2(C1(CC(C34C2=CCC5C3(C4)CCC(C5(C)C)OC6C(C(C(CO6)O)O)O)O)C)C	634.807	2.0467	1.74 × 10^−18^

**Table 6:** The pharmacokinetic properties: absorption, distribution, metabolism, excretion, and toxicity of Cimicifugoside H-2.

	Property	Value
	Water solubility	Moderately soluble
	Caco-2 permeability	No
	Gastrointestinal (GI) absorption	Low
Absorption	P-gp substrate	Yes
	P-gp I inhibitor	Yes
	P-gp II inhibitor	No
	Vd	−0.66 log L/kg
Distribution	Blood–brain barrier (BBB) permeant	No
	Fu	0.296
	CYP2D6 substrate	No
	CYP3A4 substrate	Yes
	CYP1A2 inhibitor	No
Metabolism	CYP2C19 inhibitor	No
	CYP2C9 inhibitor	No
	CYP2D6 inhibitor	No
	CYP3A4 inhibitor	No
	Clearance	0.158 log mL/min/kg (Low)
Excretion	Renal OCT2 substrate	0.698
	Skin sensitization	No
	AMES toxicity	No
Toxicity	Rat oral acute toxicity (LD50)	3.303 mol/kg
	Human hepatotoxicity	No

## Data Availability

The data that support the findings of this study are available from the corresponding author [C.A.K.] upon reasonable request.

## Abbreviations

NF-κB	Nuclear factor kappa light chain enhancer of activated B cells
IκB (α,β,ε)	Nuclear factor of kappa light polypeptide gene enhancer in B-cells inhibitor
NEMO:	Nuclear factor-κB essential modulator
IKK (1/2)	IκB kinase (alpha/beta)
IKK	Inhibitor of nuclear factor-κB kinase
PRRs	Pattern recognition receptors
TCR	T cell receptor
LTβR	Lymphotoxin beta receptor
BAFF	B cell activating factor
NIK	Nuclear factor-κB-inducing kinase
KD	Kinase domain
ULD	Ubiquitin-like domain
SDD	Scaffold dimerization domain
LZ	Leucine zipper
HLH	Helix-loop-helix
MD	Molecular dynamics
RMSD	Root Mean Square Deviation
RMSF	Root Mean Square Fluctuation
Rg	Radius of gyration
MM/PBSA	Molecular Mechanics/Poisson–Boltzmann surface area
logP	Logarithm of partition coefficient
CYP	Cytochrome P
AMES	Salmonella typhimurium reverse mutation assay
